# International Diabetes Federation guideline for management of postmeal glucose: a review of recommendations

**DOI:** 10.1111/j.1464-5491.2008.02565.x

**Published:** 2008-10

**Authors:** A Ceriello, S Colagiuri

**Affiliations:** Clinical Science Research Institute, Warwick Medical SchoolCoventry, UK; * Institute of Obesity, Natrition, and Exercise, University of SydneySydney, NSW, Australia

**Keywords:** diabetes, guideline, macrovascular, postmeal glucose, self-monitoring of blood glucose

## Abstract

Diabetes is a significant and growing concern, with over 246 million people around the world living with the disease and another 308 million with impaired glucose tolerance. Depending on the resources of different nations, intervention has generally focused on optimizing overall glycaemic control as assessed by glycated haemoglobin (HbA_1c_) and fasting plasma glucose (FPG) values. Nevertheless, increasing evidence supports the importance of controlling all three members of the glucose triad, namely HbA_1c_, FPG and postmeal glucose (PMG) in order to improve outcome in diabetes. As part of its global mission to promote diabetes care and prevention and to find a cure, the International Diabetes Federation (IDF) recently developed a guideline that reviews evidence to date on PMG and the development of diabetic complications. Based on an extensive database search of the literature, and guided by a Steering and Development Committee including experts from around the world, the *IDF Guideline for Management of Postmeal Glucose* offers recommendations for appropriate clinical management of PMG. These recommendations are intended to help clinicians and organizations in developing strategies for effective management of PMG in individuals with Type 1 and Type 2 diabetes. The following review highlights the recommendations of the guideline, the supporting evidence provided and the major conclusions drawn. The full guideline is available for download at http://www.idf.org.

## Introduction

Diabetes has no geographical boundaries and the disease, once ascribed to more affluent countries, is now known to profoundly affect developing nations [1]. More than 246 million people around the world are now living with diabetes and 308 million people are estimated to have impaired glucose tolerance (IGT) [1]. These alarming figures translate to an estimated 3.8 million people succumbing to the disease in the past year [1].

As a global issue, diabetes outcome is closely tied to the management strategies and resources available in the various regions of the world. However, even within the limitations of healthcare resources in certain nations, there is a need to optimize diabetes management to minimize related morbidity and mortality. Among the many methods recognized for improving outcome in diabetes, such as lifestyle modification and control of related risk factors, the importance of tailoring therapies to achieve specific glycaemic targets is increasingly apparent. Traditionally, intervention has largely focused on optimizing overall glycaemic control as assessed by glycated haemoglobin (HbA_1c_) and fasting plasma glucose (FPG) values [2]. However, studies have highlighted the importance of targeting postmeal hyperglycaemia and demonstrate a strong relationship between elevated postmeal glucose (PMG) and the risk of complications [3–7].

As part of its global mission to promote diabetes care and prevention and to find a cure, the International Diabetes Federation (IDF) has developed a guideline that reviews the relationship between PMG and the development of diabetes complications, as well as recommendations for the appropriate management of PMG. Below is an overview of the guideline's major conclusions and recommendations for management of postmeal glycaemia.

## Origin of guideline

The postmeal guideline was developed under the direction of the IDF, through consultation of a Steering Committee (four members) and Development Committee (14 members). These committees were composed of individuals from around the world experienced at guideline development and healthcare delivery and living with diabetes [8]. A series of four key questions served as the basis for an extensive, systematic search of the literature published over the past 20 years. Question 1: Is postprandial hyperglycaemia harmful? Question 2: Is treatment of postmeal hyperglycaemia beneficial? Question 3: Which therapies are effective in controlling postmeal plasma glucose? Question 4: What are the targets for postmeal glycaemic control and how should they be assessed? Relevant reports were obtained through a computerized search of the literature using PubMed and other search engines; scanning of incoming journals in the medical library and review of references in pertinent review articles, major textbooks and syllabi from national and international meetings on the subjects of diabetes, using relevant title and text words (e.g. postprandial, postmeal, hyperglycaemia, mealtime, self-monitoring, oxidative stress, inflammation) as search criteria. The committee reviewed over 1600 studies and numerous key reports before making recommendations based on the strength of the evidence. The draft guideline, which extended to management of both Type 1 and Type 2 diabetes, was distributed for wider review by IDF member associations, global and regional IDF elected representatives, interested professionals, industry and others on IDF contact lists.

## Rationale for postmeal glucose control

In people with normal glucose tolerance, plasma glucose generally rises no higher than 7.8 mmol/l (140 mg/dl) (which reflects the World Health Organization definition) [9]. For this guideline, postmeal hyperglycemia is defined as a plasma glucose level > 7.8 mmol/l (140 mg/dl) 2 h after the ingestion of food.

**Figure 1:**
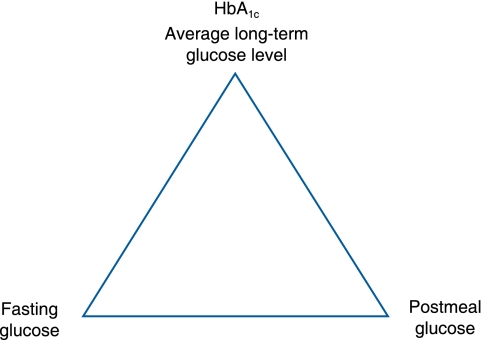
Glycated haemoglobin (HbA_1c_), postmeal glucose (PMG) and fasting plasma glucose (FPG) interrelate and are essential targets for intervention in attempts to optimize overall glycaemic control.

Development of postmeal hyperglycaemia coincides with a loss of first-phase insulin secretion, a decrease in insulin sensitivity and an inability to adequately suppress hepatic glucose production [10–12]. As frank diabetes develops [11], PMG excursions continue to worsen [13]. The contribution of postmeal plasma glucose to HbA_1c_ is proportionally greatest with HbA_1c_ values of 6.5%, while nocturnal FPG is at a near-normal level. As HbA_1c_ rises above 8%, the relative contribution of postmeal hyperglycaemia to overall glycaemic control diminishes, while the contribution of FPG predominates [13]. These results explain previous findings that, while the contribution of postmeal plasma glucose to overall glycaemia is ~70% at HbA_1c_ values < 7.3%, the postmeal contribution is ~40% with HbA_1c_ values above 9.3%[14]. Such findings form the basis for a glucose triad model of diabetes management, in which all three glycaemic parameters of HbA_1c_, PMG and FPG interrelate, and are essential targets for intervention in attempts to optimize overall glycaemic control (Fig. 1).

Postmeal or post-challenge hyperglycaemia is common in the diabetes population and is commonly found even in individuals considered to have adequate overall metabolic or glycaemic control. A cross-sectional study in the USA involving 218 individuals with Type 2 diabetes identified post-challenge glucose values ≥ 11.1 mmol/l (200 mg/dl) in nearly 74% of individuals overall and in 39% of those with HbA_1c_ values < 7.0%[15]. Individuals with Type 1 diabetes on ‘intensive’ insulin therapy are also typically subject to these glycaemic excursions, with 72-h continuous monitoring recording postmeal elevations above 7.8 mmol/l (140 mg/dl) in 77% of this patient population [16].

The closer the achieved HbA_1c_ is to the normal range, the lower the risk of complications [17,18]. Given that PMG is a major contribution to overall glycaemia across a range of HbA_1c_ values [14], targeting postmeal hyperglycaemia may help reduce risk of complications. The evidence and recommendations for the management of PMG are reviewed.

### Question 1: Is postprandial hyperglycaemia harmful?

Epidemiological studies have identified postmeal and post-challenge hyperglycaemia as independent risk factors for macrovascular disease in individuals with IGT and Type 2 diabetes [4,7,19–22]. Findings from two large-scale studies, the European ‘DECODE’ and the Asian ‘DECODA’ study, demonstrated 2-h plasma glucose to be a strong predictor of cardiovascular disease and all-cause mortality [20,21] and a better indicator than fasting glucose [21]. In addition, a meta-analysis of 38 prospective studies confirmed that, while cardiovascular risk increases linearly within a wide range of 2-h plasma glucose values, a threshold effect is detected for FPG values up to ~5.6 mmol/l (100 mg/dl) [4]. Postmeal hyperglycaemia more reliably predicted incidence of cardiovascular events in individuals with Type 2 diabetes than elevations in FPG, with cardiovascular risk greater in women than men when comparing the top vs. bottom tertiles of post-lunch plasma glucose values [19].

Along with population studies demonstrating an association between postmeal hyperglycaemia and macrovascular risk, surrogate markers of cardiovascular disease also appear to depend on PMG elevations. Even mild-to-moderate postmeal hyperglycaemia in individuals without diabetes was demonstrated to be an independent risk factor for development of carotid intima-media thickness (CIMT), a marker of atherosclerosis [7]. In addition, a causal association was found between postmeal hyperglycaemia and oxidative stress, inflammation and endothelial dysfunction [23,24] and adhesion molecules [25]. Significant reductions in myocardial blood flow and blood volume are observed following ingestion of a mixed meal in individuals with Type 2 diabetes, while such myocardial perfusion deficits are not found during the postmeal state in individuals without diabetes [26].

A number of other diabetes-related conditions are also associated with postmeal and post-challenge hyperglycemia, including independent correlation with diabetic retinopathy and neuropathy, as well as an association with diabetic nephropathy [3], increased cancer risk [27] and cognitive dysfunction in elderly individuals with Type 2 diabetes [28].

#### Recommendation

- Postmeal hyperglycaemia is harmful and should be addressed.

### Question 2: Is treatment of postmeal hyperglycaemia beneficial?

Although randomized controlled trials have not yet established whether controlling postmeal glycaemia will in itself reduce macrovascular or microvascular risk, evidence suggests that therapies ‘preferentially’ targeting postmeal hyperglycaemia appear to be beneficial [5,29,30]. Treatment with acarbose, an α-glucosidase inhibitor that delays carbohydrate absorption following meals, significantly reduced postmeal hyperglycaemia, along with risk of myocardial infarction and other cardiovascular events in individuals with Type 2 diabetes [5] and IGT [30]. A positive effect is also observed on CIMT in individuals with Type 2 diabetes with the use of repaglinide, a rapid-acting insulin secretagogue that preferentially targets PMG; after a 1-year study period, regression of CIMT (i.e. decrease in CIMT of > 0.02 mm) was observed in 52% of individuals receiving repaglinide, compared with only 18% of those treated with glibenclamide (glyburide) [29]. Similar findings are observed in individuals with IGT, demonstrating that acarbose therapy reduces CIMT progression compared with placebo control [31]. Rapid-acting insulin analogues also appear to have a positive effect on surrogate markers of cardiovascular disease, including nitrotyrosine, endothelial function and methylglyoxal (MG) and 3-deoxyglucosone (3-DG) [32–34], with acarbose treatment also showing some similar effects on these markers [35].

Along with evidence for a reduction in macrovascular risk with treatments that preferentially target PMG in individuals with Type 2 diabetes [5,29,30], findings from recent interventional studies demonstrate that targeting postmeal hyperglycaemia helps optimize HbA_1c_[6,36]. Individuals with Type 2 diabetes achieving a target HbA_1c_ ≤ 7.0% had significantly lower PMG values after the end of a 3-month intensified diabetes management programme compared with those who did not [6]. Fasting levels were not significantly different between the two groups, suggesting that, although control of FPG alone was necessary, it was not sufficient for achieving the HbA_1c_ target. In addition, severe hypoglycaemia was not associated with targeting postmeal hyperglycaemia.

#### Recommendation

- Implement treatment strategies to lower postmeal plasma glucose in people with postmeal hyperglycaemia.

### Question 3: Which therapies are effective in controlling postmeal plasma glucose?

Guidelines from various professional diabetes organizations continue to encourage physical activity, nutritional intervention and weight control as essential components of a comprehensive programme for diabetes management [37–39]. Nevertheless, the precise composition of the ‘optimal’ diet is still debated, although reducing glycaemic load (GL) has emerged as a favourable option [40,41]. Glycaemic load is an estimate of the glycaemic effects of a diet, accounting for both the type of carbohydrate consumed [i.e. according to the glycaemic index (GI)] and the amount [40]. With glycaemic load and glycaemic index reliably predicting the glycaemic response following a mixed meal [40,41], nutritional planning that incorporates these measures may not only reduce PMG excursions but is also found to modestly lower HbA_1c_[42] and possibly reduce cardiovascular risk [43].

A number of therapeutic agents that preferentially lower postmeal plasma glucose are currently available. These therapies include α-glucosidase inhibitors, glinides (rapid-acting insulin secretagogues), rapid-acting insulin, biphasic (premixed) insulins and human regular insulin. In addition, newer classes of therapies have emerged that are also associated with a reduction in PMG excursions and improvement in HbA_1c_. These agents include amylin analogues, glucagon-like peptide-1 (GLP-1) derivatives and dipeptidyl peptidase-4 (DPP-4) inhibitors [44,45] (Table 1).

**Table 1:** Therapeutic agents that preferentially lower postmeal plasma glucose and their mechanisms of action

Therapeutic class	Physiological mechanism of action
Alpha-glucosidase inhibitors	Delays carbohydrate absorption from the gastrointestinal tract
Amylin analogues	Acts as a replacement for naturally occurring amylin, a hormone secreted by pancreatic B-cells along with insulin, that decreases glucagon release, slows gastric emptying and decreases food intake
DPP-4 inhibitors	Inhibits DPP-4 enzyme that degrades GLP-1
Glinides	Stimulates a rapid but short-lived release of insulin
GLP-1 derivatives	Acts as a replacement for GLP-1, an incretin hormone secreted by the gut that stimulates insulin secretion, reduces glucagon secretion and delays gastric emptying rate
Rapid-acting insulins	Developed to mimic physiological insulin response to meals with rapid onset and peak activity and short duration of action

DPP-4, dipeptidyl peptidase-4; GLP-1, glucagon-like peptide-1.

#### Recommendation

- A variety of both non-pharmacologic and pharmacologic therapies should be considered to target postmeal plasma glucose.

### Question 4: What are the targets for postmeal glycaemic control and how should they be assessed?

A 2-h postmeal goal of < 7.8 mmol/l (< 140 mg/dl) is recommended, in line with targets published in guidelines from other associations [8,39,46]. Such guidelines define normal glucose tolerance (NGT) as post-challenge values of < 7.8 mmol/l 2 h after ingestion of a 75-g glucose load [1,37], corresponding to postmeal values of healthy individuals after meals [47]. The 2-h time frame for testing is also in agreement with published guidelines and in addition reflects the 2- to 3-h return of postmeal hyperglycaemia to basal levels in individuals with normal glucose tolerance [39,46].

The guideline encourages performing glucose monitoring as frequently as needed in order to guide therapy to achieve PMG targets. In particular, the guideline suggests considering self-monitoring of blood glucose (SMBG), as it is the only way to directly assess PMG. However, some degree of controversy surrounds SMBG use, particularly with respect to the frequency of monitoring in non-insulin requiring diabetes [48]. Nevertheless, most professional diabetes organizations continue to advocate its use [37,39,46], generally recommending that self-monitoring is performed at least three times daily in individuals treated with insulin and according to treatment regimen and level of glycaemic control in non-insulin dependent diabetes [37,38]. What is more, the use of structured SMBG appears to result in a significant reduction in HbA_1c_, even in individuals with non-insulin requiring diabetes [49]. These findings support the implementation of SMBG as part of a structured programme that may involve the training of clinicians and patients to accurately interpret results and adjust therapy accordingly in a timely manner.

#### Glycaemic goals for clinical management of diabetes

- HbA_1c_ < 6.5%
- Premeal (fasting) < 5.5 mmol/l (< 100 mg/dl)
- Two hours postmeal < 7.8 mmol/l (< 140 mg/dl)

Note: Lower glucose parameters to as near normal as safely possible. Glycaemic targets should be individualized. These goals are not appropriate for children and pregnant women.

#### Recommendations

- Two-hour postmeal plasma glucose should not exceed 7.8 mmol/l (140 mg/dl) as long as hypoglycaemia is avoided.
- Self-monitoring of blood glucose should be considered because it is currently the most practical method for monitoring postmeal glycaemia.
- Efficacy of treatment regimens should be monitored as frequently as needed to guide therapy towards achieving postmeal plasma glucose target.

## Relevance of guideline and implications

The importance of managing PMG to improve overall glycaemic control in diabetes is now fairly widely recognized in guidelines from professional associations [2,37–39,46]. As the research base continues to expand, the answers to other relevant questions are eagerly anticipated, such as whether there is a causal association between PMG and macrovascular complications and the role of SMBG in individuals who are not on insulin therapy. Until that time, logic and clinical judgment preside when interpreting the evidence presented in the guideline and deciding on an optimal management plan. Management strategies should depend on a practical evaluation of how best to integrate the recommendations into modern practice, with consideration to locally available therapies and resources.

## Conclusions

Postmeal hyperglycaemia occurs early in the development of diabetes, progressively worsens with deteriorating HbA_1c_ and is often inadequately controlled. In addition, elevations in PMG are strongly associated with a number of serious complications of diabetes, both through a direct contribution to HbA_1c_ as well as an independent association with various diabetes-related complications. Based on evidence to date, the guideline recommends implementing a comprehensive management programme that targets both postmeal and fasting glucose, which should be initiated simultaneously at any HbA_1c_ level to improve outcome in diabetes. A variety of both non-pharmacological and pharmacological therapies should be considered to target postmeal plasma glucose. Monitoring should occur as frequently as needed to guide therapy and SMBG should be considered. Subject to available therapies and resources, a 2-h postmeal plasma glucose target of < 7.8 mmol/l (140 mg/dl) is considered both reasonable and achievable.

## Comment

Since publication of the guideline, results from an interventional study of 644 outpatients with Type 2 diabetes demonstrated that the glucose spike (i.e. the difference between premeal glucose value and peak value of glycaemia during three different meals) was the most important predictor of CIMT progression compared with all examined glycaemic parameters, with peak value of glycaemia occurring 1.5 h after the start of the meal [50].

## Competing interests

AC and SC have relationships with a wide range of organizations but development of the guideline, upon which this review was based, was supported by unrestricted educational grants from Amylin Pharmaceuticals, Eli Lilly and Company, LifeScan Inc., Merck & Co. Inc., Novo Nordisk A/S, Roche Diagnostics GmbH and Roche Pharmaceuticals.

## Abbreviations

CIMT: carotid intima-media thickness
FPG: fasting plasma glucose
HbA_1c_: glycated haemoglobin
IDF: International Diabetes Federation
IGT: impaired glucose tolerance
PMG: postmeal glucose
SMBG: self-monitoring of blood glucose
